# An Integrated Care Initiative to Improve Patient Outcome in Schizophrenia

**DOI:** 10.3389/fpsyt.2015.00184

**Published:** 2016-01-08

**Authors:** Norbert Mayer-Amberg, Rainer Woltmann, Stefanie Walther

**Affiliations:** ^1^Private Practice for Psychiatry and Psychotherapy, Hannover, Germany; ^2^Private Practice for Neurology and Psychiatry, Wildeshausen, Germany; ^3^Department of Health Outcome Management, Janssen-Cilag GmbH, Neuss, Germany

**Keywords:** schizophrenia, integrated care, patient-centered, quality indicators, duration of hospital stays, psychoeducation, outpatient health care services

## Abstract

The optimal treatment of schizophrenia patients requires integration of medical and psychosocial inputs. In Germany, various health-care service providers and institutions are involved in the treatment process. Early and continuous treatment is important but often not possible because of the fragmented medical care system in Germany. The Integrated Care Initiative Schizophrenia has implemented a networked care concept in the German federal state of Lower Saxony that integrates various stakeholders of the health care system. In this initiative, office-based psychiatrists, specialized nursing staff, psychologists, social workers, hospitals, psychiatric institutional outpatient’s departments, and other community-based mental health services work together in an interdisciplinary approach. Much emphasis is placed on psychoeducation. Additional efforts cover socio-therapy, visiting care, and family support. During the period from October 2010 (start of the initiative) to December 2012, first experiences and results of quality indicators were collected of 713 registered patients and summarized in a quality monitoring report. In addition, standardized patient interviews were conducted, and duration of hospital days was recorded in 2013. By the end of 2012, patients had been enrolled for an average of 18.7 months. The overall patient satisfaction measured in a patient survey in June 2013 was high and the duration of hospital days measured in a pre–post analysis in July 2013 was reduced by 44%. Two years earlier than planned, the insurance fund will continue the successfully implemented Integrated Care Initiative and adopt it in the regular care setting. This initiative can serve as a learning case for how to set up and measure integrated care systems that may improve outcomes for patients suffering from schizophrenia.

## Introduction

Schizophrenia is a mental disorder that affects ~0.3–0.7% of people at some point in their lives (1). The chronic and disabling course of the illness may have a major impact on daily routine, quality of life, and life planning. Thus, patients need intensive and long-term support in order to be able to cope with everyday life and to lead a life as close to normal as possible.

The optimal treatment of schizophrenia patients requires integration of medical and psychosocial inputs and has to be tailored to the individual needs of the patients and their families. The patient-centered care should be provided in an outpatient setting by a multidisciplinary team and should comprise medical, social, psychological, and psychotherapeutic support (2). In Germany, various health-care service providers and institutions are involved in the process of treatment of schizophrenia. However, continuous treatment is often not possible because of the fragmented medical care system in Germany (e.g., no short-term appointments with the physician who is to provide continued treatment after discharge from hospital or no outpatient medical contact if a crisis situation sets in the evening or during the weekend) (3). To overcome these difficulties, patient outcome-oriented solutions are needed that follow an intensified, integrative approach that exceeds the provided standard care in Germany. We hypothesized that this implies the coordination of treatment options offered by different service providers to increase therapy efficiency.

### About the Integrated Care Initiative Schizophrenia

After a tender procedure, in July 2010 AOK Lower Saxony, the largest statutory sick fund in Lower Saxony, closed a contract on integrated care for schizophrenia sufferers with the Institute for Innovation and Integration in Healthcare (I3G GmbH), according to § 140 b Abs. 1 SGV V (German law on social welfare). The I3G is an independent subsidiary of the researching pharmaceutical company Janssen-Cilag GmbH. I3G bore the responsibility for the process and budget, as well as the economic risk. I3G assigned parts of the on-site operative implementation to the company Care4S GmbH. Their task was to locally build, expand, and support networks with the Integrated Care parties involved – taking up on existing structures wherever possible.

In addition, an independent multidisciplinary expert committee, composed of key stakeholders involved in schizophrenia care, such as medical specialists from hospitals and practices, experts in the areas of health care and health care research, specialist nurses, and relatives’ representatives, was established. The expert committee gave advice on the implementation, advancement, and evaluation.

### Objectives and Strategy of the Integrated Care Initiative Schizophrenia

The Integrated Care Initiative Schizophrenia pursued the goal of optimizing patient-centered care for patients suffering from schizophrenia. The initiative is based on a networked care concept that integrates various stakeholders of the health care system. Medical attention is closely linked to psychosocial support in this network. Office-based psychiatrists, specialized nursing staff, psychologists, social workers/pedagogues, hospitals, psychiatric institutional outpatient’s departments, and other community-based mental health services collaborate in an interdisciplinary approach.

In this initiative, local specialized physicians and psychiatric care services organize the outpatient treatment. However, superabundance and shortage of medical care are both to be avoided. A focus is on assisting the patients in coping with their illness in their domestic surroundings whenever possible (e.g., by home treatment). This requires close cooperation between all care providers across professional groups and institutions.

Apart from establishing and improving networks, this initiative gives patients access to an augmented range of ambulatory treatment options that are not offered or not offered to that extent in standard care in Germany. While the drug treatment options are the same as in standard care, this initiative has much more psychosocial care options. Much emphasis is placed on psychoeducation. Further efforts cover the areas of socio-therapy, visiting care, and family support. As an example, specialist nursing is available for all patients according to the needs of the patient. Home treatment is provided by psychiatric nurses directed by an office-based psychiatrist. In case of hospitalization, cooperation with clinicians is intended to ensure that inpatient treatment can be evolved into intensive outpatient treatment. Here, an expert team supports the patient. Furthermore, a 24/7 crisis service is available for patients and their families.

**Figure 1:**
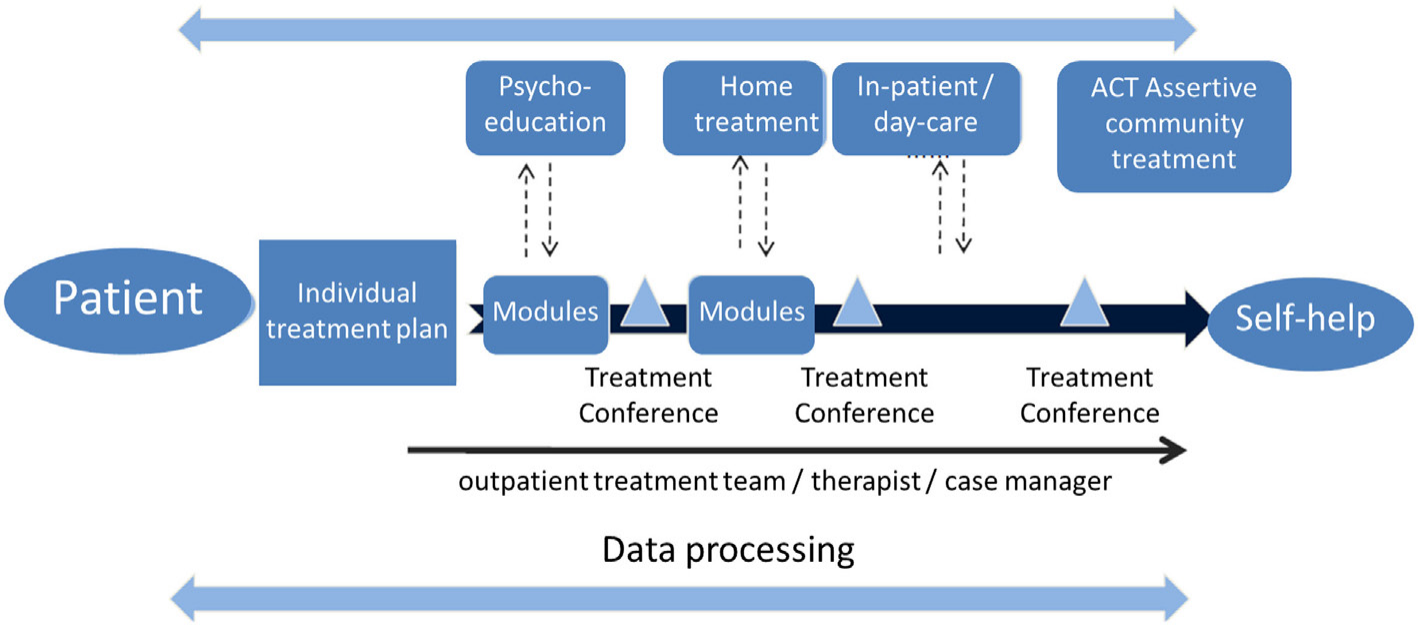
**Patient-centered approach in the integrated care initiative schizophrenia**.

The pivotal contact for the patients is the psychiatrist – he or she is in charge of the therapy. The psychiatrists are independent in their patient-oriented therapeutic decision making, as well as being committed to the current status of understanding in medical science. Therapy decisions comprise all drugs that are approved and available in the German market for the treatment of schizophrenia. Together with the other members of the care team, but above all in consultation with the patient (“shared decision-making”), the psychiatrist defines therapy modules meeting the patient’s needs. These modules correspond to the latest scientific insights by the WHO and DGPPN (German Society of Psychiatry, Psychotherapy and Neurology). They form the multilevel and modular treatment path of the Integrated Care Initiative Schizophrenia, which shall allow tailoring care to patients’ needs and personal preferences (Figure 1).

Here, we present the first experiences and results of descriptive analyses from the quality monitoring report of the Integrated Care Initiative Schizophrenia Lower Saxony during the period from October 2010 (start of the initiative) to December 2012.

## Materials and Methods

Participation in the Integrated Care Initiative was open for AOK insurees above 18 years of age with registered residence in Lower Saxony according to their health insurance card upon presentation of a confirmed diagnosis of schizophrenia (ICD-10: F20, correlating with the international statistic classification of diseases and related health problems, version 2010). The participation was voluntary. The patient had to contact a psychiatrist who was a contract partner of the Integrated Care Initiative, then he was informed about the integrated care provision and had to sign a declaration of participation. There were no exclusions regarding schizophrenia severity, comorbidities, or previous hospitalizations. In order to meet data privacy requirements, a system for data transfer was implemented.

The Integrated Care Initiative was implemented by office-based psychiatrists in cooperation with specialized psychiatric nursing staff. They introduced the patients to the various care services and types of support. Psychoeducational groups for patients and their family members were set up by the network partner, aiming at improving knowledge about the disease, which in turn enables improved compliance and early detection of relapses (4).

Routine records from the Integrated Care Initiative and accounting data of the AOK Lower Saxony as well as qualitative and quantitative effects of care recorded in an IT-documentation system served as basis for the descriptive analyses.

### Educational Materials and Training Concept

The Integrated Care Initiative provided a range of materials and documents specifically developed for conducting outpatient psychoeducation. The standardized training followed the APES manual (Arbeitsbuch PsychoEdukation bei Schizophrenie, published by Schattauer Verlag) and could be supplemented with further contents as needed.

An initiative coordinator organized workshops that provide the network partners with strategies for the implementation of psychoeducation in the individual regions. In these train-the-trainer sessions, local specialists and the outpatient psychiatric care team were educated in the theory and practice of psychoeducation based on the APES manual. Among others, the content addressed the symptoms and course of the disease, stress coping models, issues of salutogenesis and empowerment, early warning symptoms, and crisis management. Participants were qualified to conduct future psychoeducation as trainers.

The workshops on how to conduct psychoeducation took place in 1-day blocks. Didactics of knowledge transfer, visualization methods, and interactive conversation were central learning units. Role-playing exercises consolidated the theoretical knowledge.

### Quality Indicators

When establishing a new form of health care, it is essential to verifiably ensure and transparently document the quality of care. In contrast to the somatic area, in the psychiatric field quality indicators are not standard practice in Germany yet. The subjective perception of disease and concomitant personal assessment of the treatment results make valid and reliable quality measurement based on objective criteria an extremely challenging task. To face this challenge, in 2009, the Federal Association of the AOK initiated a project to develop quality indicators for patient-centered care of people affected by schizophrenia (5). The following indicators derived from this are aimed not simply at individual care areas, but rather at an integrated cross-sector treatment approach. They were used to monitor the *status quo* of the initiative:

(A) *Continuity of outpatient treatment after discharge from hospital*: the percentage of patients who received further outpatient treatment within 7 days after being discharged.
(B) *Hospital readmission rate*: the percentage of patients who were readmitted to hospital within 30 days after an inpatient psychiatric treatment.
(C) *Antipsychotic polypharmacy*: the number of patients who took at least two antipsychotics simultaneously over a timespan of at least 4 weeks during the reference period.
(D) *Compulsory treatment*: this indicator describes how many patients in the Integrated Care Initiative Schizophrenia were hospitalized due to legal requirements.
(E) *Discontinuation of treatment for more than 90 days*: the percentage of patients who had no contact with health care providers within the Integrated Care Initiative Schizophrenia for more than 90 days.
(F) *Case management*: the percentage of severely ill patients [defined by a global assessment of functioning (GAF) scale value below 50] who were in contact with a case manager during the last 6 months of the reference period.
(G) *Inclusion of relatives into the treatment*: the proportion of patients whose relatives were included in the treatment support during the reference period.
(H) *Availability of a disease self-education program*: the proportion of patients who participated in psychoeducational training during the reference period.
(I) *Number of suicides and suicide attempts*: all documented suicides and suicide attempts per 1,000 patients.

### Patient Satisfaction

In addition to analysis of the quality indicators, qualitative and quantitative effects of care from the patients’ points of view were recorded. In 2013, patients were retrospectively interviewed concerning their experience with this initiative. The patient interviews were conducted from May 1 to June 15, 2013 using a standardized eight-item questionnaire (modified ZUF-8) (6). Every answer was graded from 1 (lowest satisfaction) to 4 (highest satisfaction). Scores from all of the eight answers on the patient questionnaire were summed up to form an overall rating so that 32 was the highest rating and 8 was the lowest rating for minimum participant satisfaction.^1^

### Duration of Hospital Stays

The time that patients spent in the hospital was routinely monitored in the quality assurance review. All patients who participated in the Integrated Care Initiative for more than a year were included into a pre–post analysis of this parameter in July 2013, regardless of schizophrenia severity. The analysis compared the total number of days spent in hospital during the year before enrollment with the total number of days spent in hospital within the first year after enrollment.

## Results

By the end of 2012, 713 out of ~6,800 eligible patients were enrolled in the regions where the program was activated. The observed patient group (*N* = 713) consisted of 51% male patients and 49% female patients. The mean ages in the male and female group were 44.2 (±12.2) years and 50.3 (±12.0) years, respectively.

By the end of 2012, patients were enrolled in the Integrated Care Initiative for an average of 18.7 months. Ninety-five out of 713 patients (13.0%) had at least one hospitalization.

Data on comorbidities were available from 499 patients who participated during the period from July 2011 to June 2012. In all, 51.0 and 85.0% of the patients were diagnosed with at least one secondary psychiatric disease and at least one somatic disorder, respectively. This correlates with other epidemiological data obtained for Germany (7, 8).

### Quality Indicators

#### Continuity of Outpatient Treatment after Discharge from Hospital

More than half of the hospitalized patients (57.5%) received further outpatient treatment timely, i.e., within 7 days after being discharged.

#### Hospital Readmission Rate

In the Integrated Care Initiative Schizophrenia, a total of 18.4% of patients were readmitted to hospital within 30 days after discharge from an inpatient psychiatric treatment. International literature reports readmission rates between 20.7 and 34.5% (9).

#### Antipsychotic Polypharmacy

In the Integrated Care Initiative Schizophrenia, 21.6% of patients received two or more different antipsychotics for at least 28 days. By comparison, international literature reports 40.0% of patients with antipsychotic polypharmacy (10, 11).

#### Compulsory Treatment

Out of 95 patients who had a temporary stay at an inpatient facility, 6.3% experienced compulsory treatment.

#### Discontinuation of Treatment for over 90 Days

In the Integrated Care Initiative Schizophrenia, 15.0% of patients had no contact to a key carer for at least 90 days. Weinmann et al. give a reference value of 15.0% for this indicator (5).

#### Case Management

As no rating by GAF value was available, the analysis included not only severely ill persons but all the patients enrolled in the reference period.

In the Integrated Care Initiative Schizophrenia, 80.0% of the patients were individually looked after by the key carer system. International literature reports values between 38.0 (12) and 65.0% (severely ill patients) (13).

#### Inclusion of Relatives into the Treatment

In the Integrated Care Initiative Schizophrenia, the percentage of patients whose relatives were included into the treatment support was 17.1% (14).

#### Availability of a Disease Self-Education Program

By the end of 2012, 2.7% of the patients in the Integrated Care Initiative Schizophrenia participated in outpatient psychoeducational training. Since the launch of the initiative in autumn 2010, the model was tested in pilot regions in Lower Saxony until the first quarter of 2012. The rollout in Lower Saxony was started in April 2012. Thus, the analysis of 2012 data included only the startphase of outpatient psychoeducation, which was in the phase of implementation.

One of the first (October 2010) activated regions within the Integrated Care Initiative was Hildesheim in Lower Saxony. In this area, 8.6% of the enrolled patients took part in psychoeducational trainings by the end of 2012.

#### Number of Suicides and Suicide Attempts

As per Hor et al., the suicide rate (life-time risk) among schizophrenia patients is about 5.0% (15). In the Integrated Care Initiative Schizophrenia, the documented suicide rate amounted to 0.4% during the year 2012.

### Patient Satisfaction

**Figure 2:**
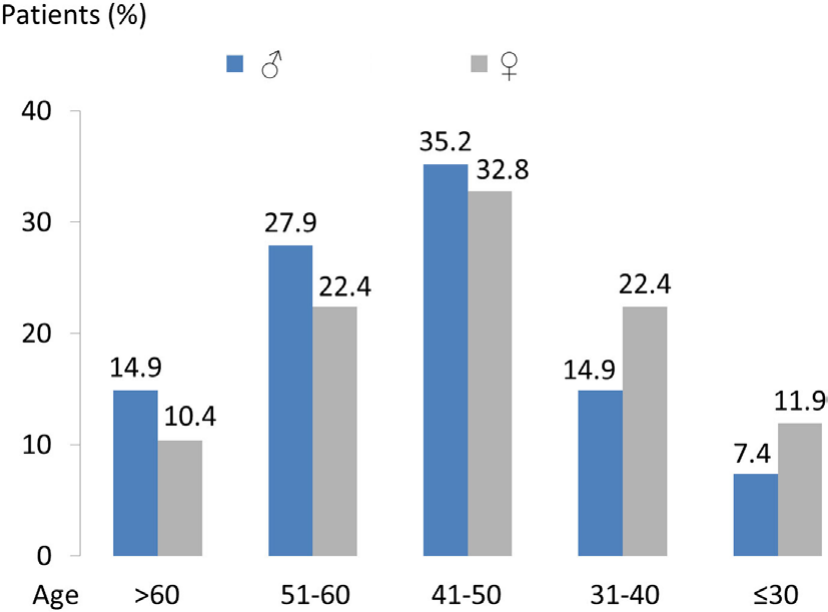
**Age and gender distribution of the 121 patients who completed the modified ZUF-8 questionnaire**.

A total of 344 patients could be identified who were registered after the start of the initiative in October 2010 and participated for at least 12 months. After a period of 1 year, patients can be expected to be fully integrated in the integrated care program and therefore be able to give a valid rating. In June 2013, 121 of the 344 patients (males: 67 and females: 54) from 25 specialist practices took part in interviews. At the time of the survey, the participating patients were enrolled in the initiative on average for almost 2 years (23.9 ± 5.5 months) and had a mean age of 47 (±12) years. Figure 2 shows the gender-specific age pattern of this population.

The findings from the interview analyses showed high patient satisfaction scores for each of the eight items of the questionnaire (Table 1). The mean total score was 27.9 (±3.2) out of a maximum of 32 points achievable.

**Table 1:** **Patient responses to the eight items of the modified ZUF-8 questionnaire**.

Item	Question	Response, *N* = 121; patients (%)			
1	Please rate the quality of the care you have received	Very good	Good	Fair	Poor
		35%	62%	1%	2%
2	Has the treatment that you have received met your expectations?	Clearly yes	Generally yes	Rather not	Clearly not
		46%	48%	3%	3%
3	To what extent did the treatment by the physician/specialist nurse service fulfill your needs?	Almost completely	By and large	Only partially	Not at all
		51%	44%	5%	0%
4	Would you recommend Integrated Care to a friend if he/she needed similar help?	Clearly yes	Probably yes	Probably not	Clearly not
		65%	26%	6%	3%
5	Please rate your satisfaction with the level of help you received from the physician/specialist nurse service within Integrated Care	Very satisfied	Satisfied	Somewhat satisfied	Dissatisfied
		50%	47%	3%	0%
6	Did the care by the physician and/or specialist nurse service help you to cope with your problems better?*	Yes, helped a lot	Yes, helped a little	No, did not help	No, has made things worse
		66%	29%	4%	0%
7	Please rate your general satisfaction with the integrated care you have received	Very satisfied	Satisfied	Somewhat satisfied	Dissatisfied
		55%	43%	2%	0%
8	Will you continue your treatment within Integrated Care?	Definitely	Probably	Probably not	Definitely not
		71%	26%	2%	1%

*One patient did not answer question 6 of the ZUF-8 questionnaire (N for question 6 = 120).

### Duration of Hospital Stay

Of the 713 patients enrolled in the Integrated Care Schizophrenia at the end of 2012, 13.3% were treated in hospital. On average, the length of hospital stays for all enrolled patients was 5.6 days. In comparison, the accumulated length of stay in 2011 for all schizophrenia patients insured by AOK Lower Saxony was 11 days.

This positive trend was confirmed by a pre–post analysis performed in 2013, which compared the sum of days spent in hospital in the year before and the year after enrollment in the Integrated Care Schizophrenia. Statistical analysis was performed in July 2013 and evaluated the data of 458 patients. The quality monitoring covered an observation period of about 2 years for the entire group.

**Figure 3:**
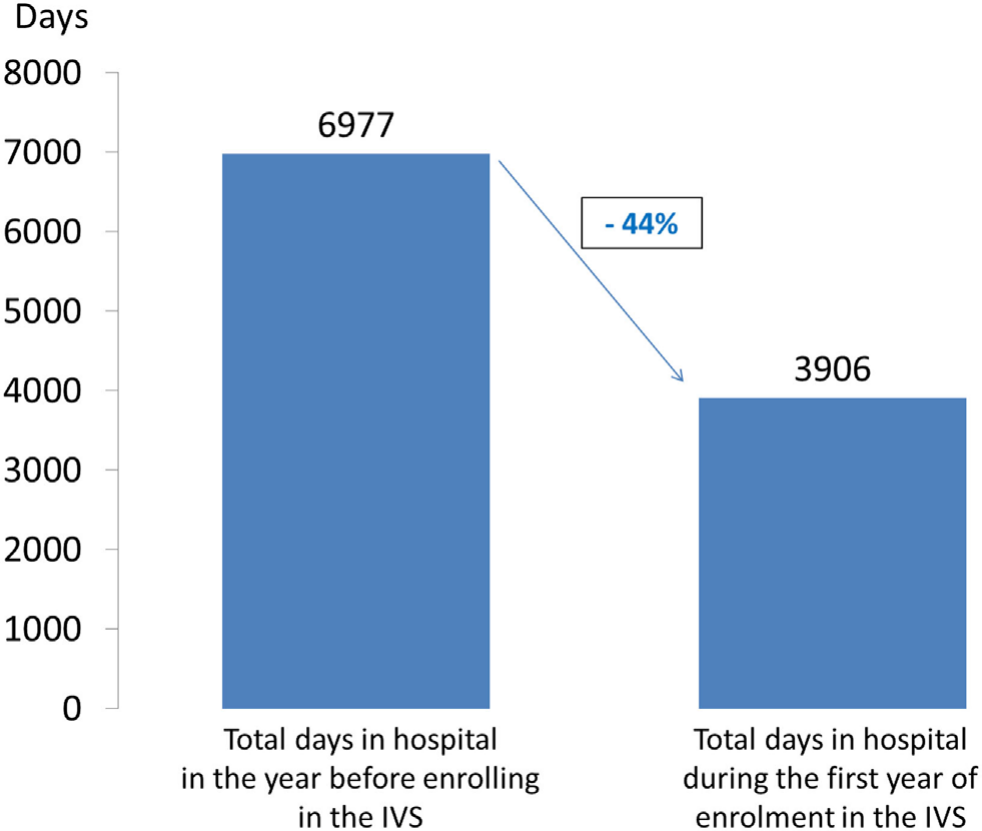
**Pre/post analysis of days in hospital due to an ICD-10-CM F20 diagnosis (*N* = 458; July 2013)**.

In the year before enrolling in this initiative, the patients spent a total of 6,977 days in hospital due to schizophrenia (F20 diagnosis). During the first year of enrollment, they spent a total of 3,906 days in hospital due to an F20 diagnosis. This means a reduction of 44.0% in the number of inpatient days during the Integrated Care Schizophrenia reference period (Figure 3).

## Discussion

Today, the routine care setting of mental ill patients remains fragmented (3). By offering more patient-centered care, the patient outcomes regarding primary, secondary, and health system parameters can be improved (16).

### The Special Role of Psychoeducation in Schizophrenia

In our opinion, a key driver was the actual implementation of psychoeducation. The Integrated Care network partners often report that the biggest obstacle is motivating the patients to participate. However, in our experience, once the patients are convinced and take part, they are enthusiastic about the psychoeducational spectrum.

Psychoeducation is defined as systematic, structured, and didactic provision of information on the disorder and its treatment. This educational method allows the afflicted and their relatives to obtain information about schizophrenia, to receive support with coping strategies, and thus to be empowered to make informed decisions concerning relapse prevention (17). There is a body of evidence supporting the fact that psychoeducation of patients with schizophrenia improves understanding of mental illness, increases quality of life, enhances compliance with antipsychotics, and can reduce relapse rates (17, 18). National and international guidelines classify the psychoeducation as a highly effective intervention in order to reduce hospital readmission rates, the resultant costs, and substantial human suffering (17).

Clinical practice shows, however, that there still exists an enormous gap between scientific findings and clinical reality. Although the benefit of psychoeducation for the empowerment of patients and the effectiveness of the therapy are proven, to date, it is provided only for every fifth inpatient with schizophrenia and only for one family member of every 50th patient (17). In the outpatient sector, psychoeducation is barely part of standard care so far. It therefore seems to be essential to offer structured psychoeducation for more patients with schizophrenia and their families than is the case today.

In the Integrated Care Initiative, the network partner himself or herself organized the training for the patients. This may be of importance because the patients confided in their caregiver. This mutual trust was essential in convincing the patients to participate.

### The Integrated Care Initiative as a Learning Case

Since the initiative started, the Integrated Care Initiative continuously developed new regions in the federal state of Lower Saxony, and, from the beginning of 2013, is now available area-wide. The first experiences with the Integrated Care Initiative in a federal state of Germany are encouraging. The introduction of the initiative improved communication between all carers. As a consequence, patients benefited from shorter waiting times, reduced bureaucracy, shorter distances, and an unchanging point of contact. The closer integration of relatives into the initiative may additionally help to better manage crisis situations. Correspondingly, patient interviews showed high patient satisfaction with the treatment path selected by the initiative.

This concept paves a way for optimizing health-care systems. Efforts and strengths of all participants who are involved in patient care could be joined successfully beyond existing structures. Linking and integrating of all the participants in the health-care system, including health-care providers, funding bodies, and health-care industry, may further improve quality of care in a sustainable manner. A health care company, in particular, can make essential contributions to achieving this goal by participating in the development and implementation of comprehensive solutions, as shown in the present Integrated Care Initiative.

Two years earlier than planned, the AOK continued the successfully implemented Integrated Care Initiative and adopted it in the regular care setting starting from January 2015. The AOK carried on ensuring optimum treatment for the insurees. The established procedures for participating physicians and psychiatric nursing services remained in place. Parallel to this, the pharmaceutical company Janssen continues to work on optimizing modular health-care concepts to increase outcomes for patients who are suffering from mental health disorders.

### Limitations and Outlook

It is important to note that the present paper is not a research study but a quality monitoring report of a novel care setting initiative. Quality monitoring of health-care performance is mandatory in the German health system (§ 140 b Abs. 1 SGV V – German law on social welfare).The data presented here were collected in the framework of the quality assurance of the integrated care project.

An evaluation designed as a prospective, observational cohort study with two independent control groups was originally planned but could not be finalized by the independent contractor due to minor recruitment numbers.

In our view, the Integrated Care Initiative can serve as a learning case for how to set up and measure integrated care systems that may improve outcomes for schizophrenic patients. Such real-world quality monitoring data are as yet rare and can make an important contribution to reflection on appropriate ways to optimize patient health-care services for mental health reasons. A further evaluation of routine data is ongoing to determine how the findings of the initiatives’ quality monitoring report will develop compared against standard care.

## Ethics

Please note: This is not a research study but a quality monitoring report of a novel care setting initiative. Quality monitoring of health-care performance is mandatory according to the German Law. The data were collected in the framework of the quality assurance of the integrated care project. Those data are rare and are often seen as an important real-life contribution to the mental health-care issue. An evaluation designed as a prospective, observational cohort study with two independent control groups was originally planned but could not be finalized by the independent contractor due to minor recruitment numbers. Further routine data analyses will compare the integrated care schizophrenia against standard care.

## Author Contributions

NM-A: significant contribution during IVS program conduction and analyses, especially on the psychoeducational content: substantial intellectual contribution to concept, revising this article critically for important intellectual content, final approval of the version to be published. RW: significant contribution to contents that refer to the independent multidisciplinary expert committee for the Integrated Care Initiative Schizophrenia: substantial intellectual contribution to concept, revising this article critically for important intellectual content, final approval of the version to be published. SW: significant contribution to all parts of the manuscript: substantial intellectual contribution to concept and analyses, writing this article, final approval of the version to be published.

## Conflict of Interest Statement

This article is sponsored by Janssen-Cilag GmbH, Neuss, Germany. However, the sponsor had no influence on the design and data collection of the described quality monitoring. Drs. Norbert Mayer-Amberg and Rainer Woltmann received honoraria as counselors for the independent multidisciplinary expert committee for the Integrated Care Initiative Schizophrenia. However, the expert committee had no influence on the design and data collection of the described quality monitoring.

## Footnotes

^1^One patient did not answer question 6 of the ZUF-8 questionnaire (*N* for question 6 = 120).
